# Fresh Cerebral Infarction-Like MRI Findings Mimicking Hyperosmolar Hyperglycemic Syndrome With Seizures

**DOI:** 10.7759/cureus.25675

**Published:** 2022-06-05

**Authors:** Toshitaka Sawamura, Kei Sawada, Ai Ohmori

**Affiliations:** 1 Department of Internal Medicine, Asanogawa General Hospital, Kanazawa, JPN

**Keywords:** fresh cerebral infarction-like lesion, mri, diabetes, seizure, hyperosmolar hyperglycemic syndrome

## Abstract

Hyperosmolar hyperglycemic syndrome (HHS) is a severe diabetes-related condition. Patients with HHS can present with abnormal magnetic resonance imaging (MRI) findings similar to those of fresh cerebral infarction. Here, we present the case of a 95-year-old woman with HHS who was initially misdiagnosed with seizures due to fresh cerebral infarction of the pons. Her MRI revealed small abnormal lesions in bilateral pons which showed hyperintensity on T2-weighted imaging and diffusion-weighted imaging. Thus, the patient was initially diagnosed with seizures associated with fresh cerebral infarction of the pons. However, hyperglycemia and hyperosmolarity were later observed, and the patient was diagnosed with HHS. Intravenous insulin and saline infusions were administered, which led to improvements in laboratory findings and seizures. The MRI findings of the pons disappeared after the treatment of HHS. Cortical restricted diffusion is observed in about 60% of cases with HHS, even if no obstruction of the artery is detected. On the contrary, patients with HHS have an increased risk of stroke during the treatment of HHS. Therefore, it is crucial for clinicians to examine patients with neurological symptoms associated with HHS not only based on MRI findings but also on neurological examination over time. In conclusion, clinicians should be aware of fresh cerebral infarction-like MRI findings in patients with HHS.

## Introduction

Hyperosmolar hyperglycemic syndrome (HHS) is an emergency diabetic condition characterized by hyperglycemia, hyperosmolarity, and dehydration without ketoacidosis. Impaired consciousness and hypotension are common symptoms of HHS. Additionally, seizures are observed in up to 25% of patients with HHS, especially in the form of focal seizures [1]. Therefore, physicians should consider HHS when considering the differential diagnosis of patients with seizures. On the other hand, fresh cerebral infarction can induce seizures. The diagnosis of fresh cerebral infarction is usually made by magnetic resonance imaging (MRI). However, patients with HHS can present with abnormal MRI findings similar to those of fresh cerebral infarction. Here, we present a case of HHS-induced seizures initially diagnosed as seizures associated with fresh cerebral infarction of the pons.

## Case presentation

**Figure 1:**
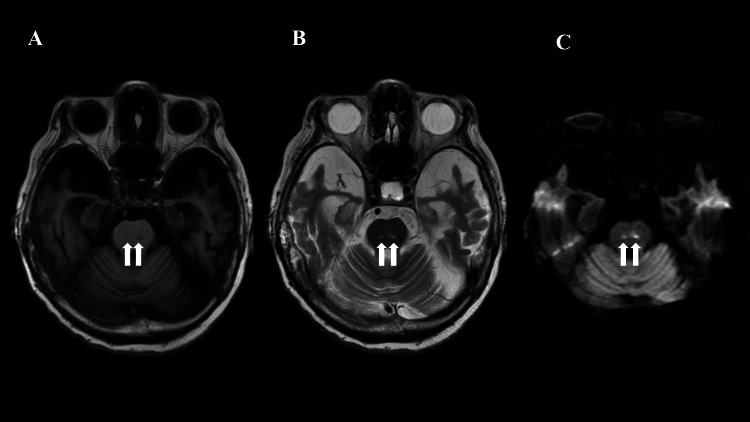
MRI findings at the time of hospitalization. Small abnormal lesions of the pons showed hypointensity in T1W (A) and hyperintensity in T2W (B) and DWI (C). T1W: T1-weighted image; T2W: T2-weighted image; DWI: diffusion-weighted image

A 95-year-old Japanese woman was transferred to our hospital for the treatment of seizures associated with fresh cerebral infarction. She had been institutionalized and had a history of hypertension, atrial fibrillation, and dementia. Eleven months prior, a gastrostomy feeding tube was created, and tube feeding was started due to loss of appetite. When tube feeding was initiated, her fasting blood glucose level and hemoglobin A1c (HbA1c) levels were 119 mg/dL and 6.3%, respectively. Five weeks prior, focal motor seizures of the right upper limb were observed for the first time and continued once every two days. She was diagnosed with epilepsy, and carbamazepine 200 mg/day was administered by her home doctor, which led to the disappearance of seizures. However, her seizures recurred three weeks after carbamazepine administration. A blood examination performed by a home doctor revealed elevated blood urea nitrogen (53 mg/dL) and creatinine (1.44 mg/dL) levels, with a high normal serum sodium level (149 mEq/L). The blood glucose level was not measured. Head MRI performed at another hospital revealed abnormal lesions in bilateral pons. The lesions were small and showed hyperintensity in T2-weighted imaging (T2W) and diffusion-weighted imaging (DWI), leading to the diagnosis of fresh brain infarction of the pons (Figure 1). The patient was transferred to our hospital for the treatment of seizures associated with a fresh cerebral infarction.

On admission, her Glasgow Coma Scale score was 9. She had a blood pressure of 87/56 mmHg and a heart rate of 102 beats/minute. Focal seizures of the right upper limb were observed. Blood examinations were not performed at the time of hospitalization. On the day of the admission, the patient had three focal seizures, and intravenous diazepam was administered each time, which led to the disappearance of the seizures. Oral carbamazepine was replaced with intravenous levetiracetam 500 mg/day. Simultaneously, intravenous ozagrel sodium and edaravone were administered for the treatment of fresh lacunar cerebral infarction.

Blood examination on day two of hospitalization showed a high blood glucose level (961 mg/dL), high HbA1c level (12.4%), hypernatremia (162 mEq/L), and hyperosmolarity (374 mOsm/L). Elevation of the urine ketone level and metabolic acidosis were not observed. The patient was diagnosed with HHS, and intravenous insulin human and saline infusions were administered. Blood glucose and serum osmolality levels improved gradually. Serum osmolality on day three, day four, and day five were 334 mOsm/L, 303 mOsm/L, and 283 mOsm/L, respectively. Seizures discontinued after the improvement in serum osmolarity. Intravenous levetiracetam was discontinued on day seven. However, the seizures did not recur. Electroencephalography after discontinuation of anticonvulsants did not reveal any abnormalities. The MRI abnormalities of the pons disappeared on MRI on day 16. Finally, her tube feeding was resumed with 12 units/day of insulin glargine and sitagliptin 50 mg/day. Her blood glucose level was controlled, and she was discharged.

## Discussion

This case of HHS was initially diagnosed as seizures associated with fresh lacunar cerebral infarction. The mechanism of seizures in patients with HHS has not yet been clearly elucidated. However, some have indicated that osmotic diuresis and dehydration damage the brain by inducing transient ischemic changes. Another indicated that the Krebs cycle is inhibited, leading to the depletion of gamma-aminobutyric acid (GABA) levels via increased metabolism, thereby lowering the threshold [2]. The most effective treatments for HHS-induced seizures are intravenous insulin and saline infusion to correct blood glucose levels and osmolality. A past review showed that 19% of patients with HHS-induced seizures were refractory to anticonvulsants [2]. However, a further 81% had certain effects of anticonvulsants. Most clinicians tend to think that HHS-induced seizures are not responsive to anticonvulsants. Thus, it is important to know that anticonvulsants also exert a certain effect on HHS-induced seizures.

In our case, the diagnosis of seizure with fresh cerebral infarction was made. The major factor for the initial misdiagnosis of HHS was thought to be a misinterpretation of MRI findings. In our case, small abnormal lesions of the pons detected as hyperintense on T2W and DWI were observed on MRI. The patient was diagnosed with seizures associated with these legions on MRI findings. However, patients with HHS can experience reversible acute cerebral infarction-like MRI abnormalities even if there is no obstruction of the artery. A review by Hiremath et al. showed that cortical hyperintensity on T2W is seen in 70% of patients with HHS. Moreover, cortical restricted diffusion is observed in 60% of cases with HHS. Despite these abnormalities being reversible, the cortical restricted diffusion can lead to a diagnosis of fresh cerebral infarction. Most reports show that HHS-induced reversible fresh cerebral infarct-like lesions spread widely in cortical and subcortical regions. There was only one report in which HHS-induced small lacunar infarction-like lesion was reported. This patient [4] was a 74-year-old man who complained of left-sided weakness and slurred speech. MRI revealed a right parieto-occipital small lesion that showed hyperintensity on T2W and DWI. The patient’s neurological symptoms fully improved with HHS treatment without the treatment of cerebral infarction. In their discussion, HHS might accentuate stroke symptoms and result in significant neurological deficit out of keeping with the size of the infarct. However, there was no description of how the abnormal findings on MRI changed after HHS treatment in this report. The MRI findings in this case are similar to those in our case. The fresh lacunar infarct-like lesions may have resolved in their case. The clear mechanisms of MRI reversible lesions in patients with HHS are unknown. However, it is theorized that either hyperosmolality or hyperviscosity results in hypoxic-ischemic injury or release of free radicals, and MRI findings occur as results of reduced energy demand from inactive brain tissue [5].

While patients with HHS can have fresh cerebral infarction-like MRI findings, they have an increased risk of acute cerebral infarction because of the presence of high blood glucose and the acute prothrombotic state [6,7]. Intermittent high glucose can promote vascular endothelial dysfunction [8]. In fact, patients diagnosed and treated for HHS sometimes develop fresh cerebral infarction during treatment even if no abnormality was detected on MRI at the diagnosis of HHS. The vascular endothelial dysfunction is mainly caused by superoxide overproduction [8]. This overproduction of superoxide and other free radicals induces the abnormalities on MRI. The abnormal MRI findings alone in a patient with HHS do not indicate fresh cerebral infarction. However, because this condition is thought to be caused by the overproduction of free radicals, it may be considered a high-risk condition for fresh cerebral infarction. The homology between this finding and cerebral infarction should be well understood.

## Conclusions

In patients with HHS, transient fresh infarction-like MRI findings can be seen. It is important to carefully monitor the neurological findings and evaluate the vascular system with various methods, rather than judging the presence or absence of MRI abnormalities alone.
